# A concept map approach to knowledge competence acquisition for student interpreters

**DOI:** 10.1371/journal.pone.0296970

**Published:** 2024-01-25

**Authors:** Dandan Sheng

**Affiliations:** School of Foreign Languages, Shanghai University of Engineering Science, Shanghai, China; Lamar University, UNITED STATES

## Abstract

As an essential component of interpreting competence, knowledge competence has long been under-researched in the field of interpreting education and training, in contrast to language competence and various interpreting skills. This study presents the results of a survey investigating student interpreters’ attitudes toward the acceptance of concept mapping as a tool to enhance their knowledge competence and examining the pathways of factors influencing these attitudes using Structural Equation Modeling (SEM). The results showed that undergraduate student interpreters enrolled in a Mandarin-English interpreting course were willing to use concept maps as a knowledge enhancement tool, and their attitudes toward this tool were positively influenced by their perceived usefulness of concept maps in supporting extra-linguistic knowledge acquisition and interpreting performance, and their perceived ease of use of concept maps. Accordingly, pedagogical implications for introducing concept maps in interpreting classes are presented.

## Introduction

Translation competence is defined as the underlying system of knowledge required to translate one written language into another [1]. It has been divided by the research group Process in the Acquisition of Translation Competence and Evaluation (PACTE) into six elements: five sub-competences (i.e., bilingual sub-competence, extra-linguistic sub-competence, translation knowledge sub-competence, instrumental sub-competence, and strategic sub-competence) and psycho-physiological components [2]. Similarly, interpreting competence, which involves oral translation into or out of a foreign language, has been broken down by researchers into elements such as bilingual (sub-)competence, extra-linguistic knowledge, and interpreting skills [3–5], and these elements are “interrelated and each is necessary for the overall macro-competence to function correctly” [6, p.91]. Of all the elements, bilingual competence and interpreting skills such as working memory have been the focus of empirical research on interpreting competence [7–9], while much less research in this regard has been done on extra-linguistic knowledge.

As interpreters face the ongoing trends of professionalization and specialization in the job market, the subject matter or domain knowledge (i.e., knowledge about a particular field of study) required by an interpreting task will increasingly influence the interpreter’s demonstration of competence and the quality of their performance in a comprehensive way, as such knowledge is a must for interpreting tasks in business, legal and technical settings. Therefore, attention to such knowledge can offer additional insights into theoretical breakthroughs in interpreting studies and promote the development of interpreting education programs at both the undergraduate and graduate levels. In response, this study aimed to introduce a concept mapping approach to the acquisition of knowledge competence by novice undergraduate student interpreters working between spoken Mandarin and English. To this end, a survey was conducted to elicit student interpreters’ attitudes regarding their acceptance of the concept map as a knowledge management tool, and the results were analyzed using path analysis via Structural Equation Modeling (SEM) to identify factors that contribute to their attitudes. Thus, the present study explores the use of concept maps to facilitate student interpreters’ acquisition of knowledge competence required by interpreter education programs with a view to preparing them for specialization as professionals.

## Literature review

### Extra-linguistic knowledge or knowledge competence

Extra-linguistic knowledge, as a crucial component of both translation competence and interpreting competence, involves various types of knowledge. In the context of translation competence, it involves general world, domain-specific, bicultural, and encyclopedic knowledge [1]. In terms of interpreting competence, it involves subject matter and thematic knowledge [3, p.9], or alternatively encyclopedic, subject matter and domain-specific knowledge [5]. In the present study, the term “knowledge competence” refers to the interpreter’s ability to satisfy his or her need for extra-linguistic knowledge, particularly subject matter knowledge, to accomplish a desired interpreting task. So far, research on translation competence and interpreting competence commonly confuses extra-linguistic knowledge with knowledge competence. Still, little empirical research in this regard has illustrated the range or amount of knowledge that is likely to constitute a translator’s or interpreter’s extra-linguistic knowledge, as well as the breadth or depth of knowledge that contributes to translation competence or interpreting competence. Under these circumstances, knowledge competence has been assumed to be either any knowledge that the translator or interpreter needs to understand the source language and form the target language, or one’s ability to recognize the need to obtain additional knowledge from external information sources to fill one’s knowledge gap in order to accomplish the translation or interpreting task [10, 11]. The latter is also referred to as “subject area competence” [6] or “subject matter competence” [11] concerning translation competence.

There is little doubt that the quality of interpreting depends to a large extent on familiarity with the background information and subject matter of the speech in the source language [4]. An empirical study conducted by Liu [12] showed that subject matter preparation has a greater impact on students’ performance in interpreting than in translating and that students with sufficient pre-task preparation can handle speeches with subject matter that is considerably unfamiliar or technical to them. Thus, Liu concluded that a thorough, in-depth understanding of the subject matter improves the quality of interpretation rather than merely a large vocabulary or a list of terminologies. Liu also found that most professional interpreters search for subject matter knowledge in addition to preparing terminology lists before an interpreting event. However, not all interpreters prepared a glossary list before working for a United Nations (UN) meeting [13]. These findings suggest that an adequate interpreting performance is achieved through the interpreter’s ability to recognize and internalize the presumably required information, i.e., the interpreter’s knowledge competence.

To date, few studies have examined how an interpreter’s extra-linguistic knowledge affects interpreting competence. Related to student interpreters working between spoken Mandarin and English, Cai and Dong [14] found that bilingual competence, as important as it is for interpreting performance, is more likely to function through the mediation of psychological competence, e.g., interpreting anxiety [15], English listening span, and Chinese speaking span. Therefore, interpreting training is likely to be a process in which students learn how to integrate and coordinate relevant competencies to perform the demanding task of interpreter programs. Although Cai and Dong focused on linguistic competencies and their information was based on a limited number of student samples, the mediating role of psychological competence highlighted the assumption that extra-linguistic knowledge is useful to student interpreters only when they recognize that such knowledge can satisfy the need to mobilize certain knowledge for target language reproduction. For example, student interpreters may actively process their extra-linguistic knowledge if they find it helpful in identifying essential terms in the source message and, if they do not know all their meanings, deciding how to reproduce the fewest but necessary meanings of the terms in order to make the message meaningful.

In summary, despite the consensus on the importance of extra-linguistic knowledge for interpreting performance and quality, there has been a lack of interpreting research on knowledge competence. As a result, few studies have examined such areas as how knowledge and knowledge competence differ, what kind of knowledge is required and how much it matters [16], what effect knowledge has on the quality of interpreting and to what extent, and so on. However, it is worth noting that knowledge is not the same as knowledge competence when it comes to interpreting. Extra-linguistic knowledge can only be important if the interpreter can activate it to fill the knowledge gap and perform an interpreting task. Therefore, it is knowledge competence, rather than unlimited knowledge or any set(s) of knowledge, that is an essential component of interpreting competence. More specifically, knowledge competence may include the ability to identify, acquire, and apply the necessary extra-linguistic knowledge to complete a task. In addition, there is a clear need for a practical model of competence for student interpreters similar to the empirically supported model of translation competence. Until such a model is available, research into the various component competencies is also desirable, especially for the least researched area of competence in question.

### Acquisition of knowledge competence

Because knowledge competence has long been understudied, its acquisition has been similarly neglected and even bypassed in many undergraduate and graduate interpreting programs, which focus students primarily on mastering cognitive processing skills for transcoding (form-based approaches) or sense-making (meaning-based approaches) [17, p.56, 18, 19, p.384]. Under such circumstances, knowledge competence is not taught but acquired by students who gradually make connections between pre-task preparation efforts and improvements in classroom interpreting performance. Their efforts typically involve online searches for domain knowledge and the preparation of bilingual glossaries [19, p.128, 140]. Gile [3] proposed three stages of ad hoc knowledge acquisition (namely the acquisition of information for a specific interpreting task) for conference interpreters, i.e., advance preparation, last-minute preparation, and in-conference knowledge acquisition. As for the training of student interpreters, Gile called for separate knowledge acquisition exercises and demonstrations as well as constant attention to the quality of ad hoc knowledge acquisition. However, he seemed to advocate the advantage of terminological preparation (e.g., glossary preparation) over extra-linguistic knowledge preparation before an interpreting assignment [3, p.147], and noted that terminological preparation seems to be more widespread among non-teaching practitioners, while extra-linguistic knowledge preparation is advocated in the literature by theoreticians and teachers [3, p.146].

Despite limited research on the acquisition of knowledge competence for student interpreters, lessons can be drawn from the curricula of specialized translation courses in German and Danish translation programs with high demands on the knowledge acquisition of student translators. Kastberg [11] proposed two common approaches to knowledge acquisition for student translators in translation programs within the two countries. One approach requires students to complete prerequisite courses, such as Introduction to Technological Science or Fundamentals of Technological Science, before enrolling in a specialized course in Sci-tech translation. The other approach mandates that students take relevant courses within the same discipline on which the specialized translation course is based before enrolling in the specialized translation course. For example, a student must take courses such as International Business, Business Communication, or International Trade before enrolling in a Business Translation course. Arguments about the former approach involve how to identify the basic knowledge of a discipline and how to teach students to apply this range of acquired knowledge in the classroom to the translation of texts with specific subject matter. Arguments about the latter include how to decide on the selection and practicality of disciplinary areas for teaching, and how to determine the appropriate breadth or depth of knowledge in such areas. In both cases, however, there is a risk that students’ attention will be diverted from language and translation to knowledge of a specific domain. Moreover, no research has yet investigated the effects of student translators’ knowledge on their translation competence and the quality of their translations of specialized texts.

### Concept mapping and its applications in specialized translation courses

Developed by Joseph Novak in the late 1970s, concept mapping has been widely used to teach and evaluate science courses and educational programs. Concept mapping is a technique for visualizing structured knowledge about a core topic by linking related concepts and labeling their relationships. It reflects the user’s ability to organize knowledge, construct meaning, and generate new knowledge. The steps in creating a concept map are as follows: (1) searching for concepts related to a topic previously known or unknown to the user; (2) specifying the relationships between the concepts found; and (3) drawing by hand or using concept mapping software to represent these concepts and their relationships. The two key elements in constructing a concept map are the relationships or links between the concepts and the propositions that contain two or more concepts linked together to form a meaningful statement [20].

Concept mapping serves as an effective strategy to help students learn meaningfully by making explicit connections between concepts [21]. Previous research suggests that students in college-level science tutorials find that the use of concept mapping enhances meaningful learning of topics by linking related concepts [22]. The tool also reveals the user’s knowledge gaps and misconceptions, which may lead to alternative conceptions that the assessor can identify and address [23]. In this way, concept maps function not only as a pedagogical tool but also as a means of formative assessment. Relational and structural scoring methods have been used to evaluate concept maps [24–26]. The former focuses on the accuracy of logical relationships between concepts but overlooks the quantity and organization of knowledge; the latter emphasizes the structure of concepts and the relationships between different branches of knowledge. In general, the greater the number of valid links between concepts, the more sophisticated the map is assessed to be. Logistically, the former requires less knowledge on the part of the evaluator, so it is relatively easy to apply pedagogically.

Kastberg [11] and Kastberg and Ditlevsen [27] took the initiative to introduce concept maps as a knowledge tool in specialized translation courses to assess students’ abilities to mobilize their cognitive abilities to acquire knowledge. The teaching process involves the following four steps. First, the teacher informs the students about the topic of the upcoming translation task and asks them to create a concept map on this topic following the standards that the teacher has designed. Second, the teacher evaluates the concept maps or the students evaluate each other’s concept maps. Third, the teacher provides the students with the translation text to translate. Finally, the teacher and students re-evaluate the usefulness of concept maps after the translation task is finished. Kastberg [11] pointed out that the acquisition of knowledge competence is much more important than the acquisition of knowledge, and the introduction of concept maps establishes a link between translation teaching and students’ knowledge management skills, thus promoting the lifelong learning potential of prospective professionals.

To address the lack of research on the acquisition of knowledge competence among student interpreters, the present study aims to apply concept maps to the acquisition of knowledge competence among undergraduate student interpreters working between spoken Mandarin and English and to conduct a survey to investigate their attitudes toward the acceptance of concept maps as a tool for enhancing such competence and the factors that influence their attitudes. Hence, the following two questions are explored in this study:

1. What are student interpreters’ attitudes toward using concept maps to acquire subject matter knowledge to complete the interpreting task?
2. What are the factors that influence their attitudes?

## Methods

### Concept maps

The current study proceeded with the teacher introducing concept maps as a knowledge tool for undergraduate student interpreters working between spoken Mandarin and English to help them relate and integrate their prior knowledge with newly presented knowledge from the teacher about the subject matter of an assigned interpreting task informed beforehand by the teacher. The teacher may, based on the assigned interpreting task, offer special advice on adding valuable concepts needed to fulfill the knowledge gap related to the interpreting task. Students received ninety minutes of training, including instructions on hand-made concept mapping techniques and demonstrations of master maps [20], before they were asked to produce concept maps for the subject matter of an assigned interpreting task and make an oral presentation based on their maps. Students were instructed to produce the map by hand rather than using the software so that they could create the map without access to computers in the classroom.

### Concept map scoring

**Fig 1:**
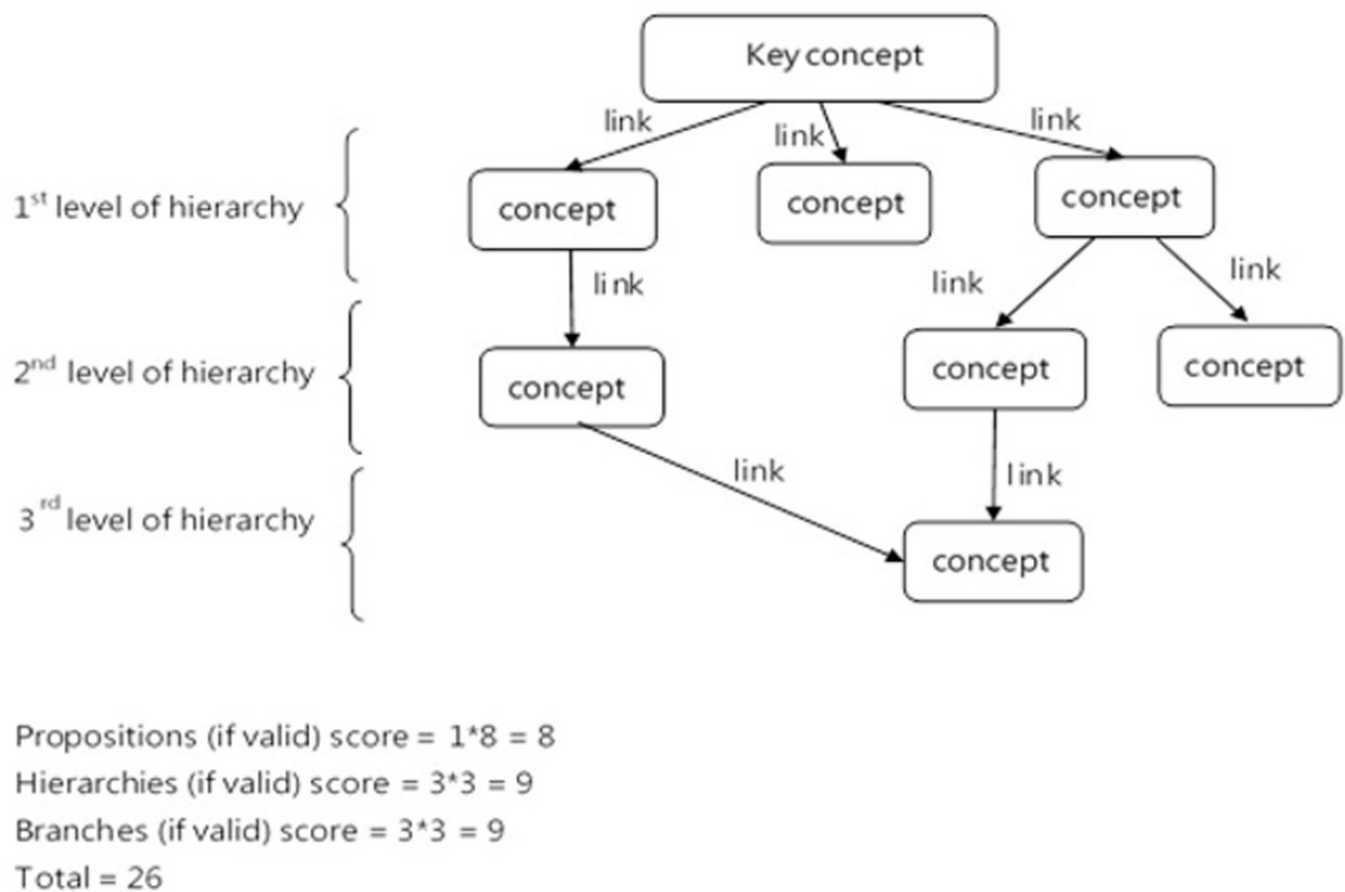
Instructions for the structural scoring method.

Students’ concept maps were then scored using a scoring method adapted from the structural scoring method proposed by McClure, Sonak et al. [26]. The final map score was determined by summing the scores of the propositions, hierarchy levels, and branches of the first-level hierarchy identified on the maps (Fig 1). Specifically, one point was assigned to each valid proposition, defined as two concepts connected by a labeled arrow indicating the relationship between the concepts; three points were assigned to each valid hierarchy level, defined as branching structures showing superordinate-subordinate categorical relationships between concepts; and three points were assigned to each valid branch of first-level hierarchy concepts (i.e., the branch score is equal to three multiplied by the number of valid links of the first-level hierarchy). Possible scores ranged from 0 to 100. A sample of 20 concept maps was scored by the researcher who doubled as the teacher-assessor and a second rater who was informed of the scoring method. The two assessors received several rounds of training and reached a consensus on general guidelines suggesting rating strategies to be used. Finally, a total of 112 candidates’ concept maps were assessed by the two assessors to offer final consensus ratings and the inter-rater agreement was measured by Spearman’s rho, which was significant at the 0.01 level at .965.

### Concept map revisions

Students were then allowed to revise their maps according to the teacher’s feedback on their maps and presentations. After undertaking all these pre-task preparations involving concept maps, students were asked to complete the assigned interpreting task, which required them to interpret the audio recording of the source speech in Mandarin delivered by a native speaker into English and vice versa on the assigned topic. The purpose of this series of tasks was to help the student interpreters identify their need for subject matter knowledge, acquire such knowledge through concept mapping activities, and apply the newly acquired knowledge to the interpreting task. After the students completed the two interpreting tasks one after the other aided by concept mapping activities, a survey was administered and the survey data were subjected to factor analysis.

### Participants

The study was submitted to the academic ethics committee of Shanghai Second Polytechnic University and deemed exempt from review as the survey data accessed were anonymous and did not allow identification by the author of individual participants. The participants of this survey were 112 full-time Chinese undergraduate English majors enrolled in the mandatory Mandarin-English Interpreting course at Shanghai Second Polytechnic University, China, in 2014. The course spanned two successive semesters and the participants were near the end of the second semester when participating in the survey. Among them, male students accounted for 7.2% and female students accounted for 92.8%. After completing interpreting tasks supported by concept mapping in four sessions (about 12 class hours) during the semester, they were asked to fill out the tailored survey entitled “Student interpreters’ Perceptions of Using Concept Maps”. The response rate was 100%.

### Survey

The survey design was primarily based on Davis’s Technology Acceptance Model (TAM) [28], which has been widely used to predict user acceptance of technology use [29]. Although participants created concept maps by hand, the adoption of TAM was supported by the potential application of concept map software in interpreting classrooms in the future. The design incorporated the model’s two core constructs, perceived usefulness and perceived ease of use. Given the research questions of the present study, perceived usefulness was further divided into two components: perceived usefulness of concept maps in acquiring subject matter knowledge and perceived usefulness of concept maps in improving interpreting performance. Additionally, the survey included students’ awareness of the importance of subject matter knowledge and their attitudes toward using concept maps to acquire subject matter knowledge, two dimensions designed to help elicit students’ attitudes toward the acceptance of concept maps as a tool to enhance knowledge competence in interpreting classes. In summary, the survey was designed to examine the following five dimensions: (1) students’ awareness of the importance of subject matter knowledge, (2) perceived usefulness of concept maps in acquiring subject matter knowledge, (3) perceived usefulness of concept maps in improving interpreting performance (hereafter referred to as perceived usefulness for interpreting), (4) perceived ease of the use of concept maps (hereafter referred to as perceived ease of use), and (5) their attitudes toward using concept maps to acquire subject matter knowledge (hereafter referred to as attitudes toward concept maps).

The survey also consisted of personal information about the subject and a 5-point Likert scale ranging from strongly disagree (1), disagree (2), uncertain (3), agree (4) to strongly agree (5). For a pilot test, item analysis was conducted, and a total of 16 items and 1 personal information question were included in the final version of the survey. The 16-item survey was administered to 112 students (see Table 2). The appropriateness of factor analysis of the survey data was determined using the Kaiser-Meyer-Olkin (KMO) measure of sampling adequacy (0.94) and Bartlett’s test of sphericity (120, p<0.001). A KMO above 0.60 and a significant value of Bartlett’s test of sphericity are considered adequate for factor analysis [30, 31]. Factorial validity was assessed using principal components analysis and orthogonal rotation. The resulting five-factor solution was consistent with the above-mentioned five dimensions designed for the survey, explaining 72.43% of the total variance. The reliability test showed that the internal consistency, as measured by Cronbach’s α coefficients, was satisfactory for the five factors, i.e., importance awareness of subject matter knowledge (.684), perceived usefulness for subject matter knowledge (.782), perceived usefulness for interpreting (.821), perceived ease of use (.715), and attitudes toward concept maps (.902). The test indicated a high degree of construct validity and reliability of the survey.

### Data analysis

Descriptive analysis and Pearson correlation analyses were first used to examine the relationships among the five factors included in the study. Stepwise regression was then used to examine the best predictors of student interpreters’ attitudes toward concept maps. Since neither correlation nor regression analysis could reveal causal relationships among all five factors, an exploratory analysis was performed with SEM via AMOS 20.0 and the path coefficients were analyzed to test a fit model. Conformity assessment of the model was calculated by Chi-square (x^2^), goodness-of-fit test, P, RMSEA, GFI, AGFI, NFI, and CFI values for the data.

## Results

### Importance awareness of subject matter knowledge

According to the summary of results in Table 1, the results on importance awareness of subject matter knowledge indicate that students seemed to be highly aware of the importance of subject matter knowledge in improving interpreting performance (mean = 4.44, SD = .47). There is a moderate and significant positive correlation between students’ importance awareness of subject matter knowledge and their attitudes toward concept maps (r = .382, p < .01) (see Table 1), suggesting that their positive attitudes toward concept maps are associated with the usefulness of concept maps in facilitating their preparation of subject matter knowledge.

**Table 1:** The summary table of importance awareness of subject matter knowledge, perceived usefulness for subject matter knowledge, perceived usefulness for interpreting, perceived ease of use, and attitudes toward concept maps on descriptive statistics and correlation coefficients (n = 112).

No.	Dimension	Mean	SD	1	2	3	4	5
1	Importance awareness of subject matter knowledge	4.44	0.47	1				
2	Perceived usefulness for subject matter knowledge	3.97	0.65	.411**	1			
3	Perceived usefulness for interpreting	3.9	0.67	.292**	.568**	1		
4	Perceived ease of use	3.63	0.7	.299**	.500**	.426**	1	
5	Attitudes toward concept maps	3.81	0.81	.382**	.675**	.591**	.505**	1

*: p < .05

**: p < .01

***: p < .001

Based on Table 2, 93.7% of the students believed that possessing subject matter knowledge helped improve interpreting performance (Question #1). An overwhelming 94.6% of the students believed that the teacher should teach them how to acquire subject matter knowledge (Question #2). Additionally, over 99% of the students recognized the great importance of pre-task knowledge preparation for unfamiliar subject matter (Question #3).

**Table 2:** A survey of student interpreters’ perceptions of using concept maps (n = 112).

Dimension	No	Item	Strongly disagree (1)	Disagree (2)	Uncertain (3)	Agree (4)	Strongly agree (5)
Importance awareness of subject matter knowledge	1	Acquiring knowledge about the subject matter of an interpreting task in advance improves my interpreting performance.	0.0%	0.0%	6.3%	49.1%	44.6%
	2	The teacher should instruct the students on how to acquire subject matter knowledge.	0.0%	2.7%	2.7%	51.8%	42.9%
	3	When the topic of the source language speech is unfamiliar, I need to prepare the subject matter knowledge.	0.0%	0.0%	0.9%	39.3%	59.8%
Perceived usefulness for subject matter knowledge	4	Making a concept map helps me to acquire subject matter knowledge about a topic that I am already familiar with.	0.0%	6.3%	8.0%	63.4%	22.3%
	5	Making a concept map increases the breadth of my subject matter knowledge.	0.0%	5.4%	15.2%	56.3%	23.2%
	6	My classmate’s oral presentation according to his or her concept map enriches my knowledge of the subject.	1.8%	4.5%	17.0%	58.0%	18.8%
Perceived usefulness for interpreting	7	Making a concept map related to the subject matter of the source language speech improves my interpreting performance.	0.0%	5.4%	14.3%	64.3%	16.1%
	8	The oral presentation exercise in English according to the concept map improves my fluency in the corresponding interpreting task.	0.0%	9.8%	10.7%	58.9%	20.5%
	9	The oral presentation exercise in English according to the concept map improves my accuracy in the corresponding interpreting task.	0.0%	8.0%	12.5%	61.6%	17.9%
Perceived ease of use	10	I have fully mastered the method of making a concept map.	0.9%	25.0%	33.0%	36.6%	4.5%
	11	My concept map can demonstrate the breadth of my subject matter knowledge.	0.0%	6.3%	14.3%	57.1%	22.3%
	12	My concept map can demonstrate the depth of my subject matter knowledge.	0.0%	12.5%	19.6%	48.2%	19.6%
Attitudes toward concept maps	13	I like to use the concept map as a method of acquiring subject matter knowledge.	4.5%	8.9%	10.7%	56.3%	19.6%
	14	Creating a concept map is an effective way to acquire subject matter knowledge.	2.7%	6.3%	13.4%	57.1%	20.5%
	15	Creating a concept map is worth the time.	1.8%	11.6%	14.3%	58.9%	13.4%
	16	I would like to know about software tools for making concept maps.	0.9%	12.5%	6.3%	53.6%	26.8%

### Perceived usefulness for subject matter knowledge and perceived usefulness for interpreting

Considering the results of the perceived usefulness for subject matter knowledge in Table 1, students tended to agree with the usefulness of concept maps in acquiring subject matter knowledge (mean = 3.97, SD = .65), and their perceived usefulness for subject matter knowledge was most highly correlated with their attitudes toward concept maps (r = .675, p < .01), indicating that student interpreters’ perceived usefulness of concept maps for subject matter knowledge had the greatest effect on their attitudes toward concept maps. According to Table 2, it is found that 85.7% of the students believed that when they were assigned a familiar subject to interpret (Question #4), making a concept map before the interpreting task could help them gather wanted subject matter knowledge easily. The findings indicated that the students were able to use concept maps properly both to prepare for a familiar topic and to expand their subject matter knowledge (Question #5). Moreover, 76.8% of the students thought that they could gain useful information from listening to other mappers’ presentations (Question #6).

On the other hand, concerning the perceived usefulness of concept maps for interpreting, the survey showed that students tended to agree with the usefulness of concept maps for improving interpreting performance (mean = 3.90, SD = .70), and such perception was significantly correlated with their attitudes towards concept maps (r = .591, p < .01), next to the perceived usefulness of concept maps for subject matter knowledge. Results from Table 2 illustrated that 80.4% of the students believed that concept mapping led to the improvement of their interpreting performance (Question #7). Both 79.4% and 79.5% of the students believed that the oral presentation according to the concept map could help improve the fluency and accuracy of their interpreting performance respectively (Questions #8 and #9).

### Perceived ease of use

The results of the survey showed that students appeared positioned between “uncertain and agree” (mean = 3.63, SD = .70), suggesting that the students were not ideally proficient in the use of concept maps. According to Table 2, 41.1% of the students believed that they had already fully mastered concept mapping techniques, while 25% of the students disagreed, and 33% of the students were unsure (Question #10). However, 79.4% and 67.8% of the students believed that the map they drew could represent the quantity and quality of their subject matter knowledge respectively (Questions #11 and #12), indicating that they could realize the effectiveness of concept maps.

### Attitudes toward concept maps

The results of the survey showed that student interpreters tended to agree with the introduction of concept maps in interpreting classes (mean = 3.81, SD = 0.81), and their attitudes were significantly correlated with all the other four factors as discussed above (p < .01). In Table 2, a total of 75% of the students were willing to use concept maps to acquire subject matter knowledge (Question #13). Altogether 77.6% of the students believed that concept mapping was an effective approach to acquiring subject matter knowledge (Question #14). As high as 72.3% of the students agreed that their time spent on concept maps was rewarding (Question #15). Furthermore, 80.4% of the students were willing to try using concept mapping software for similar assignments (Question #16). It appeared that the students would be interested in being equipped with electronic concept mapping tools, and the introduction of concept mapping software would have a positive effect on their acceptance of concept maps in interpreting classes in the future.

### Factors affecting students’ acceptance attitudes

After the descriptive and correlation analyses, the survey data were subjected to stepwise regression analysis to determine the best linear combination of importance awareness of subject matter knowledge, perceived ease of use, perceived usefulness for interpreting, and perceived usefulness for subject matter knowledge for predicting students’ acceptance attitudes. The regression method of “enter” showed that the combination of the four independent variables significantly predicted students’ acceptance attitudes (F(3,112) = 42.337, p < .001), with all of them significantly contributing to the prediction (p < .05) except importance awareness of subject matter knowledge (p>.05). The R^2^ value from stepwise regression analysis was 0.54, which indicated that 54% of the variance in students’ attitudes toward concept maps was explained by the model.

Perceived usefulness for subject matter knowledge was significantly predicted by three factors, i.e., perceived usefulness for interpreting, perceived ease of use, and importance awareness of subject matter knowledge (F(3,112) = 28.871, p < .001), with all of them significantly contributing to the prediction (p < .05). The R^2^ value from stepwise regression analysis was 0.445, which indicated that 44.5% of the variance in students’ perceived usefulness for subject matter knowledge was explained by the model.

**Fig 2:**
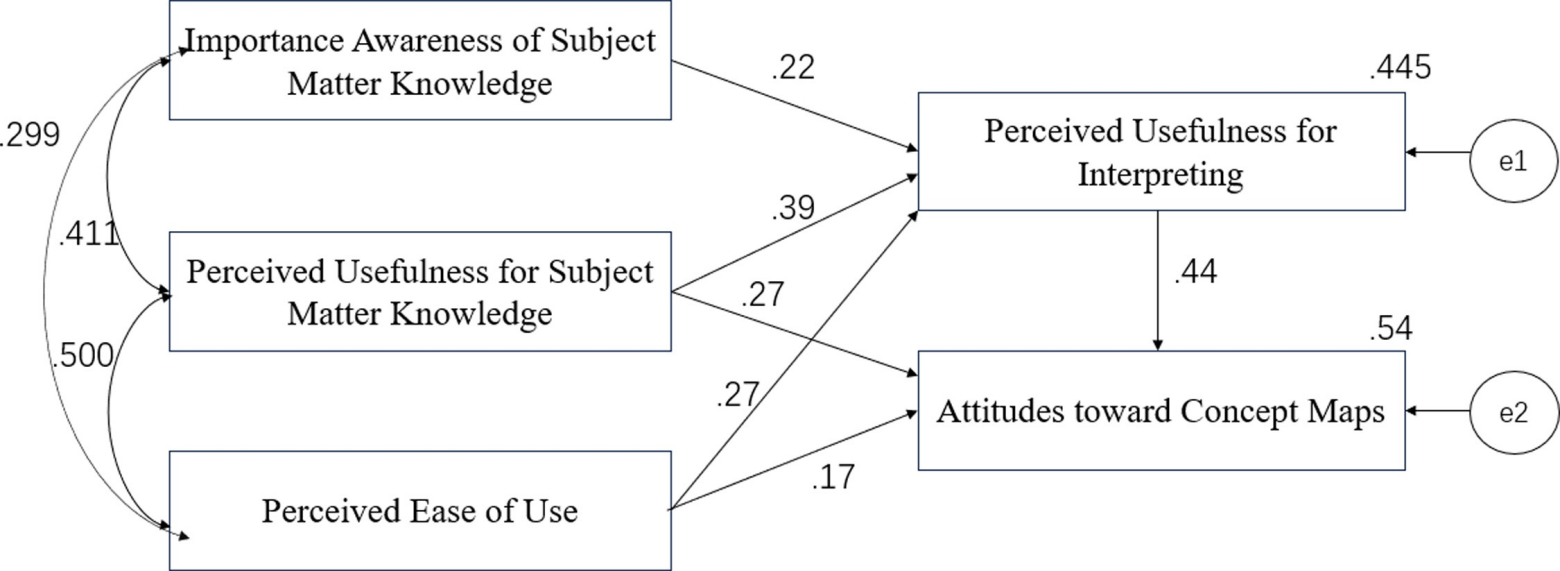
Pathways of student interpreters’ acceptance attitudes toward concept maps.

Finally, SEM was used for path analysis to explore causal relationships among the five factors involved. An exploratory pathway analysis was performed with SEM via Amos 20.0 and the path coefficients were analyzed to test a fit model. The output of the specification search in Amos provided the fit measures, and finally, a statistically satisfactory model was obtained (χ^2^ = 1.521, DF = 1, P = 0.217, RMSEA = 0.069, GFI = 0.995, AGFI = 0.918, NFI = 0.963, CFI = 0.983). A summary of the path coefficients of the factors was also generated to show the direct effect and indirect effects of each factor involved (see Fig 2).

As shown in Fig 2, students’ perceived usefulness of concept maps had the strongest effect on their attitudes. Specifically, perceived usefulness of concept maps for subject matter knowledge had the largest direct effect on attitudes toward concept maps (.44), and perceived usefulness of concept maps for interpreting had an equally strong total effect (.44) with its direct effect on students’ attitudes toward concept maps (.27) plus its indirect effect through the moderator of perceived usefulness for subject matter knowledge (.17). In comparison, perceived ease of use had a total effect (.29) with its direct effect (.17) plus its indirect effect (.12) on students’ attitudes toward concept maps, ranking it next to perceived usefulness. Somewhat surprisingly, importance awareness of subject matter knowledge had no direct effect and only a small indirect effect (.096) on attitudes toward concept maps through the moderator of perceived usefulness for subject matter knowledge.

## Discussion

The present study found the pathways of student interpreters’ acceptance attitudes toward concept maps based on the data from a survey tailored for undergraduate student interpreters. The survey examined five dimensions to find an effective approach to developing student interpreters’ knowledge competence, an important component of their interpreting competence. The results of the survey revealed students’ awareness of the importance of subject matter knowledge, perceived usefulness of concept maps for subject matter knowledge, perceived usefulness of concept maps for interpreting performance, perceived ease of use of concept maps, and attitudes toward concept maps.

The results for students’ awareness of the importance of subject matter knowledge corresponded with an empirical study that discovered the positive effects of students’ preparation of subject matter knowledge on their consecutive interpretations from French into English [12]. Other studies [32, 33] also found that student interpreters could benefit greatly as well as professional interpreters from prior subject matter knowledge to interpret simultaneously specialized speeches from Spanish into English. These studies suggested that students needed to use prior knowledge not only to interact with the knowledge presented during formal instruction [34], but also to construct new knowledge by relating it to relevant concepts they already possess [35] in order to perform both general and specialized interpreting tasks.

For student interpreters, their awareness of the importance of prior subject matter knowledge is raised probably because such knowledge helps them not only to understand information conveyed in the speech but also to make meaningful input when they do not understand all the detailed information. In addition, their prior knowledge may be very different from what the teacher assumes for college students [36]. In this case, if the teacher arranges few activities for knowledge preparation or gives students insufficient time to prepare for an interpreting topic that the teacher considers familiar to them, students may underperform to varying degrees. Their underperformance may not be caused by their inadequate interpreting competence but by their lack of adequate knowledge about the topic. Thus, there is an urgent need for student interpreters to improve their knowledge competence [12, 19, p1].

In the present study, student interpreters’ perceived usefulness of concept maps was approached from two perspectives, i.e., their perceived usefulness of concept maps for subject matter knowledge and their perceived usefulness of concept maps for interpreting. The results on the former showed that as student interpreters were generating maps by connecting concepts, students seemed to gain a deeper understanding of the collected and connected concepts as well as their relationships [21, 22]. Meanwhile, they could cultivate their ability to make logical connections between different branches of knowledge presented on the map.

Findings also seemed to imply that students’ prior subject matter knowledge could affect the extent to which the students could efficiently use concept maps as a tool to acquire new knowledge. It was discovered in class that the teacher could assist students by providing reference materials on the topic if the topic was unfamiliar for students to interpret. Under this circumstance, students could easily find concepts related to the subject matter and might shorten the time for making a concept map. Additionally, students may create concept maps that are more comparable to one another, which would make it easier for them to refer to each other’s maps and for the assessor to evaluate them.

The present study also suggested that the teacher should adjust the scoring method based on how specialized the source speech is for the students to interpret. The scoring method can give different weights to the number of concepts, links, branches, hierarchies, etc. so that students can decide to focus more on the breadth or depth of subject matter knowledge when generating their maps. Therefore, the teacher should develop a scoring method that best fits the objectives of the concept mapping exercise and can reduce the evaluation workload as much as possible [26, 37].

Further, students’ perceived usefulness of concept maps for subject matter knowledge may be considered as a result of an innovative process of collaborative learning through concept maps. It has been reported that concept mapping supports collaborative learning [38]. In this study, the concept map presentation exercise was designed for the student mappers to better absorb the knowledge they mapped and the knowledge they shared about each other’s maps. The ultimate purpose of this exercise was to increase the input and integration of students’ subject matter knowledge so that their knowledge system was advanced to help facilitate the fulfillment of knowledge competence.

On the other hand, the perceived usefulness of concept maps for interpreting performance partly depended on the teacher’s design of the evaluation method for concept mapping. The oral presentation task was arranged for students to further assimilate subject matter knowledge represented in the concept map and improve their fluency in addressing a specialized topic [39]. The teacher then evaluated the concept map and provided advice on how to expand the subject matter knowledge by adding more relevant concepts or formulating more accurate propositions based on the collected concepts and their relationships during the presentation. As students in the survey tended to acknowledge that these interrelated exercises were useful in improving interpreting performance, they were very likely to recognize the close connection between the ability to acquire subject matter knowledge and the ability to apply such knowledge to meet their knowledge gap and interpreting needs. Thus, they would feel more motivated to enhance their knowledge competence for the benefit of interpreting performance.

Additionally, student interpreters’ perceived usefulness of concept maps for interpreting performance may be explained by the advantage of concept mapping in effectively reducing students’ anxiety before and during a task [15]. With reduced anxiety levels before or during the interpreting task, students may be able to fully utilize their psychological competence, which contributes to their interpreting performance. Therefore, the process of drawing a concept map could serve student interpreters as an effective means of pre-task preparation, the quality of which can be easily checked due to the concept map’s feature of knowledge visualization.

The results of the perceived ease of use showed that college students should have no difficulty in mastering the mapping technique since concept maps have been widely used in elementary and secondary education. However, the results imply that students need more training hours to fully master mapping techniques for conceptual organization and integration, such as organizing levels of hierarchy, labeling links, and optimizing layout. In light of this, the teacher is suggested to assign relatively easy, familiar topics for concept mapping in the initial phase of this type of exercise, or to provide master maps for demonstration. However, the latter seems to risk limiting how students organize their concept maps [40]. Alternatively, the teacher can model the construction of concept maps, but students may value the opportunity to spend time constructing concept maps on their own or in small groups [22].

The results of student interpreters’ attitudes towards concept maps demonstrated their strong willingness to invest time and effort in concept mapping for similar exercises in the future. Overall, the survey results indicated that student interpreters tended to have a positive perception of the advantage of using concept maps as a knowledge tool for the benefit of interpreting performance. Such perceptions may enable them to promote their ability to use concept maps for knowledge acquisition and to advance their knowledge competence and, subsequently, interpreting competence.

### Factors affecting students’ acceptance attitudes

The current study worked out a model of pathways of student interpreters’ acceptance attitudes toward concept maps. The purpose of ascertaining such attitudes was to advance students’ awareness of the importance of extra-linguistic knowledge and knowledge competence for the benefit of interpreting competence. The model was consistent with the expectation that perceived usefulness and perceived ease of use had significant effects on users’ attitudes [28] toward concept maps as knowledge management tools. First and foremost, student interpreters’ acceptance attitudes toward concept maps were significantly attributed to their perceived usefulness of concept maps for acquiring subject matter knowledge and for improving interpreting performance. Because the pre-task preparation through tailored activities surrounding concept maps served to promote students’ knowledge competence, leading to their enhanced interpreting performance. While pre-task preparation among student interpreters and professionals commonly centers around glossary or terminology preparation [13, 32, 33], preparation for subject matter knowledge attempts to address the most overlooked component of students’ interpreting competence. Following perceived usefulness, perceived ease of use was found to influence students’ acceptance attitudes directly and indirectly via perceived usefulness. These two causal pathways are supported by the notion that lower cognitive burden frees up students’ attentional resources, thereby allowing students to focus on other learning matters [40]. On the other hand, students’ importance awareness of subject matter knowledge could hardly predict their attitudes toward knowledge management tools used for pre-task preparation. This may indicate that students’ acceptance attitudes toward any techniques and methods aimed at knowledge competence needed to be subject to experiments in interpreting education.

## Conclusions

The present study conducted a survey and adopted SEM to find out that introducing concept maps into the interpreting classroom at the tertiary level was considered acceptable by student interpreters. Also, their attitudes of acceptance were significantly and positively influenced by their perceived usefulness and perceived ease of use of concept maps, although their perception of the importance of subject matter knowledge in interpreting performance had little effect on their attitudes.

Thus, implications for interpreter education are clear. First, the introduction of concept maps explores a new approach to acquiring knowledge competence that is neither overly focused on interpreting skills nor on knowledge of a specific domain. The approach can help to address the most overlooked component of student interpreters’ competence. Second, the method of integrating concept maps into interpreting education needs to be elaborated to fully achieve the effectiveness of the tasks underpinned by concept maps. The dual purpose of concept mapping activities is facilitating students’ knowledge acquisition and promoting their knowledge competence for the benefit of interpreting competence. Therefore, in the present study oral presentations based on the concept map were required to facilitate students’ knowledge acquisition and improve their oral fluency for subsequent interpreting tasks. Also, the study may suggest letting students experience an improvement in their interpreting performance. For example, their interpreting performance can be tested before and after their participation in related concept mapping activities. Finally, the use of concept mapping software can be welcomed in an interpreting class. Such software may help to increase the ease of use of concept maps for students and reduce the assessment workload for teachers. Future studies might survey students about how they perceive the benefits of sharing their concept maps with peers and how participants use teacher feedback to improve and apply their concept maps to the interpreting task. More studies would also be helpful in examining the utilization of concept maps for knowledge competence. One such study could look at the correlation between students’ concept map scores and their interpreting performance to determine the extent to which subject matter knowledge and knowledge competence affect interpreting performance.

## Supporting information

S1 Table Summary for multivariate regression analysis over attitudes toward concept maps. (DOC)

S2 Table Summary for multivariate regression analysis over perceived usefulness for subject matter knowledge. (DOC)
