# Supplementary material for: A concept map approach to knowledge competence acquisition for student interpreters
Source: PLoS One. 2024 Jan 25;19(1):e0296970. doi: 10.1371/journal.pone.0296970 (PMC10810455; doi:10.1371/journal.pone.0296970)
Supplement: S1 Table — (DOC) [file pone.0296970.s001.doc]

S1 Table. Summary for Multivariate Regression Analysis over Attitudes toward Concept Maps

| Variables | | R | R^2^ | adjusted R^2^ | F | Beta | t | Sig. |
| --- | --- | --- | --- | --- | --- | --- | --- | --- |
| Dependent | Attitudes toward concept maps | 0.735 | 0.54 | 0.528 | 42.337 *** |  |  |  |
| Independent | Perceived usefulness for subject matter knowledge |  |  |  |  | 0.435 | 5.151*** | 0.001 |
|  | Perceived usefulness for interpreting |  |  |  |  | 0.27 | 3.343*** | 0.001 |
|  | Perceived ease of use |  |  |  |  | 0.172 | 2.240* | 0.027 |

*p<0.05, *** p<0.001
