# Supplementary material for: A concept map approach to knowledge competence acquisition for student interpreters
Source: PLoS One. 2024 Jan 25;19(1):e0296970. doi: 10.1371/journal.pone.0296970 (PMC10810455; doi:10.1371/journal.pone.0296970)
Supplement: S2 Table — (DOC) [file pone.0296970.s002.doc]

S2 Table. Summary of Multivariate Regression Analysis over **Perceived Usefulness for** Subject Matter Knowledge

| Variables | | R | R^2^ | adjusted R^2^ | F | Beta | t | Sig. |
| --- | --- | --- | --- | --- | --- | --- | --- | --- |
| Dependent | Perceived Usefulness for Subject Matter Knowledge | 0.667 | 0.445 | 0.43 | 28.871 *** |  |  |  |
| Independent | Perceived Usefulness for Interpreting |  |  |  |  | 0.39 | 4.834*** | 0.001 |
|  | Perceived ease of use |  |  |  |  | 0.269 | 3.332*** | 0.001 |
|  | Importance awareness of subject matter knowledge |  |  |  |  | 0.216 | 2.821** | 0.027 |

*p<0.05, *** p<0.001
